# An insight into the mechanism of charge-transfer of hybrid polymer:ternary/quaternary chalcopyrite colloidal nanocrystals

**DOI:** 10.3762/bjnano.5.137

**Published:** 2014-08-08

**Authors:** Parul Chawla, Son Singh, Shailesh Narain Sharma

**Affiliations:** 1 CSIR-Network of Institutes for Solar Energy (NISE), National Physical Laboratory, Dr. K.S. Krishnan Road, New Delhi, 110012, India

**Keywords:** chalcopyrites, charge-transfer, hybrid organic-inorganic, nanocomposites, P3HT

## Abstract

In this work, we have demonstrated the structural and optoelectronic properties of the surface of ternary/quaternary (CISe/CIGSe/CZTSe) chalcopyrite nanocrystallites passivated by tri-*n*-octylphosphine-oxide (TOPO) and tri-*n*-octylphosphine (TOP) and compared their charge transfer characteristics in the respective polymer: chalcopyrite nanocomposites by dispersing them in poly(3-hexylthiophene) polymer. It has been found that CZTSe nanocrystallites due to their high crystallinity and well-ordered 3-dimensional network in its pristine form exhibit a higher steric- and photo-stability, resistance against coagulation and homogeneity compared to the CISe and CIGSe counterparts. Moreover, CZTSe nanocrystallites display efficient photoluminescence quenching as evident from the high value of the Stern–Volmer quenching constant (*K*_SV_) and eventually higher charge transfer efficiency in their respective polymer P3HT:CZTSe composites. We modelled the dependency of the charge transfer from the donor and the charge separation mechanism across the donor–acceptor interface from the extent of crystallinity of the chalcopyrite semiconductors (CISe/CIGSe/CZTSe). Quaternary CZTSe chalcopyrites with their high crystallinity and controlled morphology in conjunction with regioregular P3HT polymer is an attractive candidate for hybrid solar cells applications.

## Introduction

Organic photovoltaic (OPV) devices composed of polymer matrices can be regarded as promising third-generation solar cells amongst emerging PV technologies owing to their unique mechanical flexibility for tailored applications [1–2]. A variety of non-expensive techniques for the processing of OPVs such as dip-coating, screen printing, ink-jet printing etc. has added to their versatility [3–4]. However, as compared to conventional inorganic solar cells, the performance of OPVs is often restricted by low carrier mobility issues. The emergence of hybrid solar cells is based on the concept of promoting carrier mobility in OPV systems by the incorporation of inorganic semiconductor materials as electron acceptors into organic photovoltaics [5]. Here, a charge-separation at donor–acceptor heterojunctions is a key process, which takes center stage in determining the energy conversion efficiency of hybrid photovoltaics. Hybrid solar cells enjoy an advantage of intrinsically high carrier mobility, which is caused by inorganic materials dispersed in organic matrices. Inorganic materials with unique properties such as an enhanced absorption with varied particle sizes due to quantum confinement effect, relatively high electron mobility, high surface area and good thermal stability can provide an alternative path for the development of organic photovoltaics [5–6]. To this day, numerous inorganic semiconductors such as ZnO, TiO_2_, CdSe, CdS, PbSe and PbS have been studied. Particular focus has been put on the investigation of selenides or sulfides in conjunction with organic polymers (MEH-PPV, P3HT) for hybrid solar cell applications [6–7]. However, the toxicity and hazardous issues of inorganic materials, in particular of Cd and Pb, poses a serious threat to the environment. This has limited the realization of hybrid solar cell systems for commercialization [7]. On the other hand, copper indium diselenide (CISe) and related materials in conjunction with the conjugated polymers are characterized by the capability to act as effective electron acceptors. Furthermore, they have the unique ability to achieve an enhanced performance, so that they are also beneficial for nanocomposite-based solar cells [8–9]. There are several advantages of using this class of materials with polymers such as high absorption coefficient (~10^5^ cm^−1^), high reproducibility, high efficiencies and good stability [8–10] in comparison to the other inorganic–organic nanocomposites based devices. The superior characteristic properties of chalcopyrite based semiconductors and their optical energy gap engineering, which is tunable within the solar spectrum, renders CISe and related materials a very promising PV material in the near future. However, to the best of our knowledge, investigations of CISe and related nanocrystal-polymer based solar cells are scarce. A notable study reports on a combination of poly(2-methoxy-5-(3,7*-*dimethyloctyloxy)-1,4-phenylene vinylene) (MDMO-PPV) and poly(3-hexylthiophene) (P3HT) with (CISe) and copper indium disulfide (CIS) nanocrystals involving a bulk heterojunction device prepared from copper indium disulfide and p-doped poly(3,4-ethylenedioxioxythiophene):poly(4-styrenesulfonate) complex (PEDOT:PSS) [11–12]. Recently, nanocrystals of one of the well-known quarternary chalcopyrite copper-zinc-tin-selenide (CZTSe) has been receiving considerable attention as a promising candidate for low-cost active absorber layers as it displays similar structure and optical properties to CISe. In order to achieve an efficient hybrid solar cell performance, it is imperative to control the morphology of both organic and inorganic components without any phase separation at macroscopic scale. The implementation of such a control of the morphology crucially depends on a combination of highly crystalline and defect-free inorganic CISe and related materials as an acceptor layer and an ordered morphology and well-crystalline nature of P3HT polymer as a donor layer. In the present work, we have dispersed TOPO-capped CISe/CIGSe/CZTSe nanocrystals of varied crystallinity into a P3HT polymer matrix. Successive washing of CISe-related nanocrystals in a mixture of methanol and toluene was employed to ensure the adequate removal of insulating TOPO/TOP-ligands and thus allowing for an efficient interaction between the inorganic nanocrystals and the polymer. The structural and optical properties of the resulting hybrid organic–inorganic composites were studied in detail. The mechanism of charge generation and separation across the polymer–chalcopyrite semiconductor interface has been depicted in consideration of the results obtained from various complimentary characterization techniques.

## Results and Discussion

**Figure 1:**
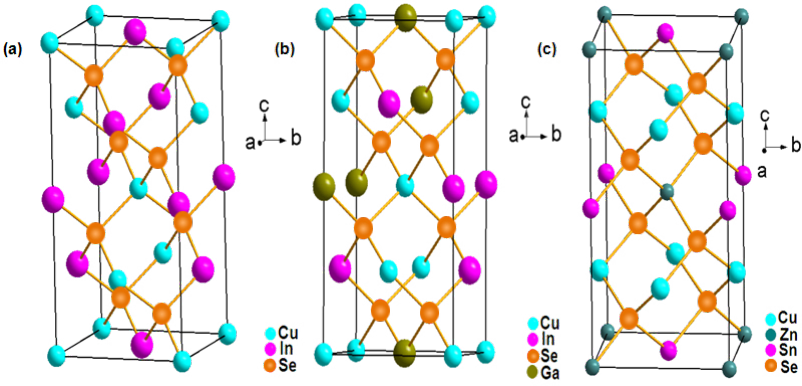
The crystal structures of (a) CISe, (b) CIGSe and (c) CZTSe.

CISe is known to acquire a chalcopyrite lattice structure, which usually is a diamond-like structure similar to the sphalerite structure. The difference lies in the ordered substitution of the Cu and In element on the Zn sites of the sphalerite resulting in a tetragonal unit cell as shown in Figure 1a. CIGSe exhibits a tetragonal chalcopyrite space lattice with space group I42d as shown in Figure 1b. Its lattice structure is almost similar to the cubic lattice structure where each Cu atom, In or Ga atom is bonded to four Se atoms in a tetrahedral fashion and, in turn, the Se atom is tetrahedrally coordinated to two Cu atoms and two In and Ga atoms. This tetrahedral coordination refers to the covalent bonding between group I, III and VI elements, which results in a sp^3^ hybridization. However, along with the covalent nature in bonding an ionic bonding character is present [13]. Therefore, the group-I and group-III metals, i.e., Cu, In and Ga, are frequently denoted as cations, whereas the group-VI Se atoms are denoted as anions in the context of explaining the detailed crystal structure of these chalcopyrites. CZTSe usually exists in the form of kesterites and stannites where every group III element (In or Ga) in the chalcopyrite lattice structure is replaced by a group II and group IV element, i.e., Zn and Sn. This sustains that the octet rule is satisfied upon displacement of these atoms (shown in Figure 1c).

**Figure 2:**
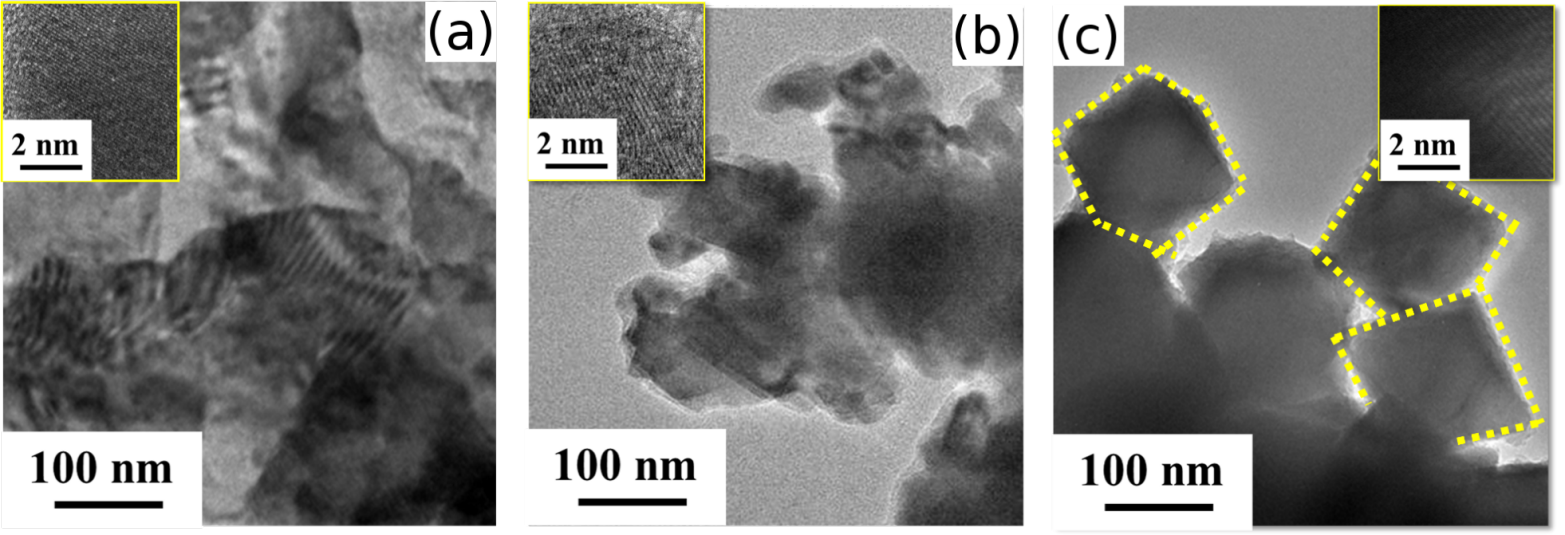
TEM Micrographs of (a) CISe nanocrystals, (b) CIGSe nanocrystals and (c) CZTSe nanocrystals. The insets depict HRTEM.

An investigation of the morphologies of the synthesized nanocrystals was carried out by transmission electron microscopic (TEM) studies. The corresponding TEM micrographs of CISe, CIGSe and CZTSe nanocrystals are depicted in Figure 2. Figure 2a shows the TEM image of CISe nanocrystals of the size of 70–80 nm characterized by tetragonal morphologies, more agglomeration in the nanocrystals, and lack of the distinct presence of the isolated nanocrystals. The inset in Figure 2a shows a high resolution TEM (HRTEM) image, which demonstrates the presence of crystalline planes with an interplanar spacing *d* of 0.34 nm. Figure 2b shows the TEM micrograph of CIGSe nanocrystals with a size of 100–120 nm and exhibiting a slight improvement in the appearance of the tetragonal morphology, which is characteristic of these chalcopyrites-based nanocrystals. The inset depicts the HRTEM micrograph with well-aligned crystalline planes and an enhanced crystallinity in comparison to its Ga-deficient counterpart; i.e., CISe. On the other hand, CZTSe shows a superior manifestation of morphology as evidenced by its distinct tetragonal nanocrystals (Figure 2c). Although slight overlap of nanocrystals is still present, an improvement in terms of crystallinity can be seen due to the emergence of nanocrystals of the size of 150–200 nm. The HRTEM micrograph shown as an inset in Figure 2c depicts the presence of sharp crystalline planes with an interplanar spacing of 0.325 nm.

**Figure 3:**
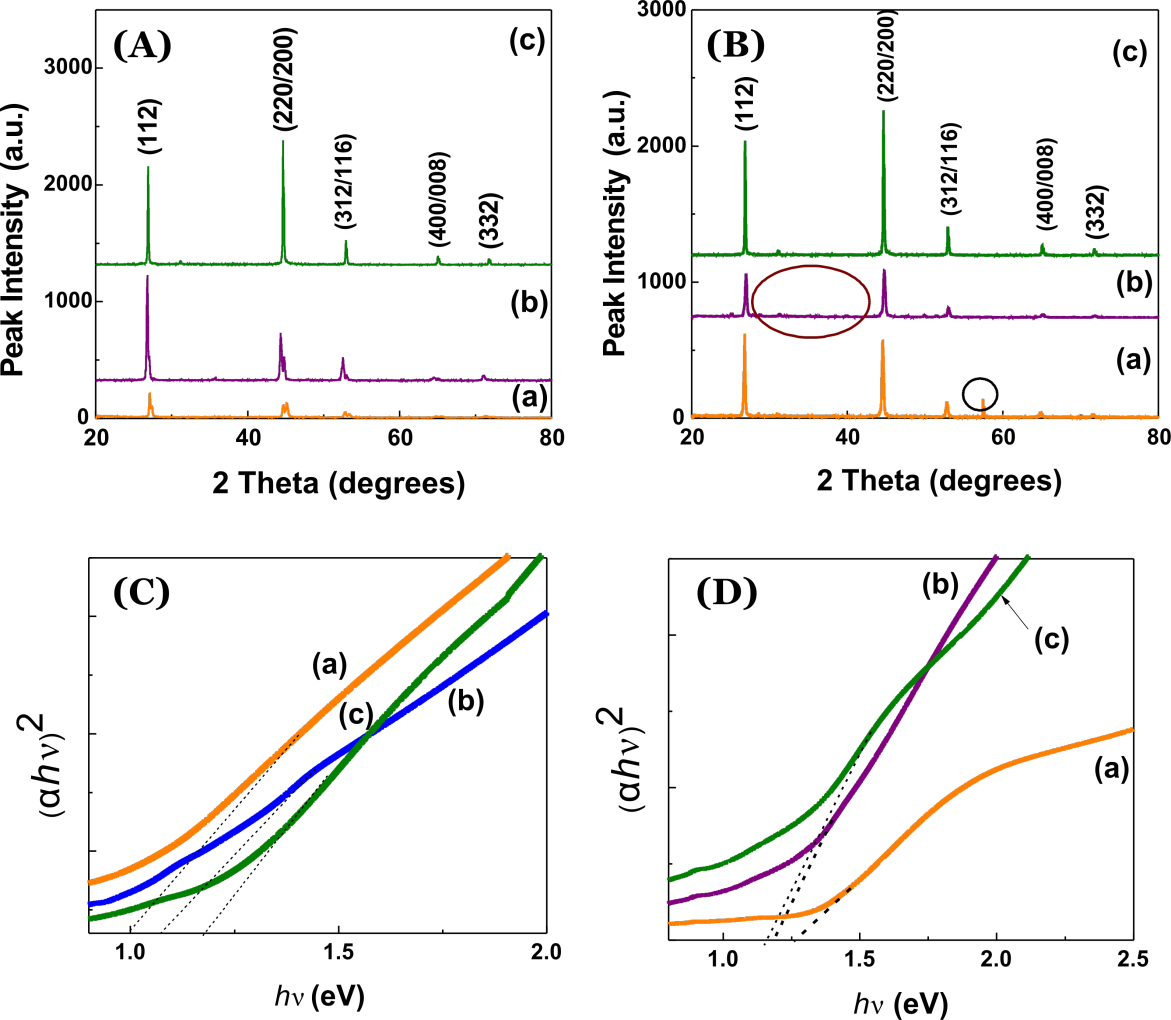
X-ray diffraction pattern of (A) pristine and (B) light-soaked, optical band gap determination based on Tauc’s plot, (α*h*ν)^2^ vs *h*ν plot of (C) pristine (D) light-soaked chalcopyrite nanocrystals of (a) CISe, (b) CIGSe and (c) CZTSe.

However, a similar trend was observed upon light-soaking studies that were carried out to investigate the stabilities of the as-synthesized nanocrystals of CISe, CIGSe and CZTSe. Light-soaking or extended light illumination studies indicate the existence of stable phases and the sustaining ability upon exposure to light as well as the associated heating effect. Here, both aid in the determination of the most stable phase among these set of chalcogenides. The light-induced degradation in CISe-based material tends to modify the properties with a light exposure for several hours. In our case, we carried out light-soaking experiments on spin-casted thin-films for CISe, CIGSe and CZTSe with an exposure time of almost one week (~168 hours). Possible property changes of the nanocrystals were investigated by X-ray diffraction (XRD) studies and UV–vis–NIR spectroscopy. Figure 3A displays the XRD pattern of pristine CISe, CIGSe and CZTSe nanocrystals characterized by the presence of prominent peaks of (112), (220/200), (312/116), (400/008) and (332). Figure 3B shows the X-ray diffraction patterns for the light-soaked CISe, CIGSe and CZTSe nanocrystals. As evident from Figure 3, CISe, CIGSe and CZTSe nanocrystals exhibit the formation of phase-pure compounds with CZTSe featuring the purest phase formation. Figure 3B(a) depicts the light-soaked CISe and shows the the emergence of an extra peak (encircled). Figure 3B(b) shows the XRD pattern for light-soaked CIGSe nanocrystals and small extra peaks can be observed. Figure 3B(c) depicts the XRD pattern of light-soaked CZTSe and there is no remarkable appearance of a noisy pattern or small peaks. Since no annealing-induced defects upon light-soaking are created in CZTSe, this observation points to a high stability of the formed CZTSe phase compared to the CISe and CIGSe phases. A similar trend can also be seen in Figure 3C(a–c), which shows the enhancement of the optical bandgap (as calculated from the Tauc’s plot involving the absorption coefficient, α and the photon energy *h*ν) from CISe to CZTSe. The bandgap values of pristine CISe, CIGSe and CZTSe are ≈1.03 eV, 1.1 eV and 1.15 eV, respectively (Figure 3C(a–c)). As can be observed from Figure 3D(a–c), the enhanced bandgap values for CISe, CIGSe and CZTSe are 1.26 eV, 1.18 eV and 1.15 eV, respectively. During prolonged light exposure there might occur a reorientation and reformation of bonds depending on the stability of the nanocrystals. Thus, based on the findings outlined above one can infer that the order of stability of different chalcopyrite nano-structures upon light-soaking is CZTSe > CIGSe > CISe.

**Figure 4:**
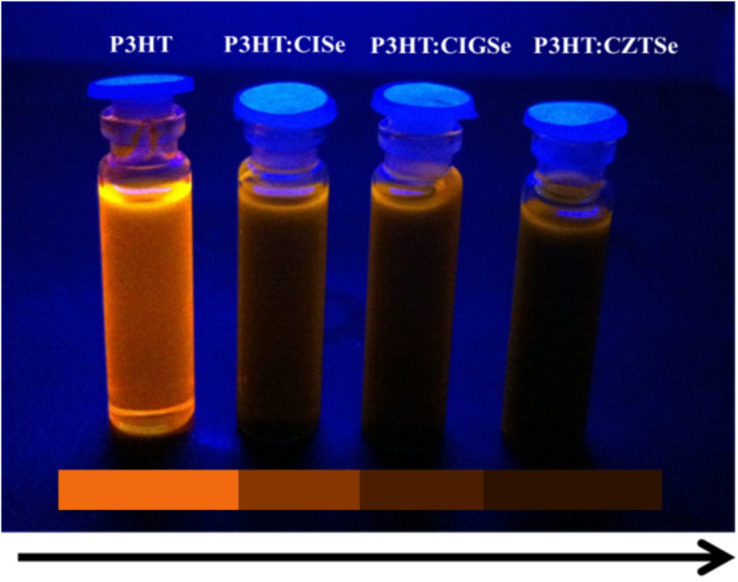
UV-lamp exposed photographs of polymer P3HT, P3HT:CISe, P3HT:CIGSe and P3HT:CZTSe nanocomposites.

Based on these results, we studied the interaction between the polymer and each class of synthesized nanocrystals. The detailed data and the charge transfer capabilities have been discussed as follows. Figure 4 shows photographs of the polymer P3HT and the polymer nanocomposites of P3HT:CISe, P3HT:CIGSe and P3HT:CZTSe nanocrystals in toluene exposed to a UV lamp. A clear color gradation from left to right is visible. The first one shows the image of the toluene dispersed P3HT, which is highly luminescent compared to the samples. From left to right nanocomposites for the same concentrations of CISe, CIGSe and CZTSe are getting darker.

This is due to the varying ability of the nanocrystals from CISe to CZTSe to reduce the luminescence intensity of the polymer to a considerable extent. The nanocrystals which lower the luminescence of the polymer the most are the best charge-transfer material from the polymer to nanocrystals. In our case, the best charge transfer capability is found for CZTSe nanocrystals.

**Figure 5:**
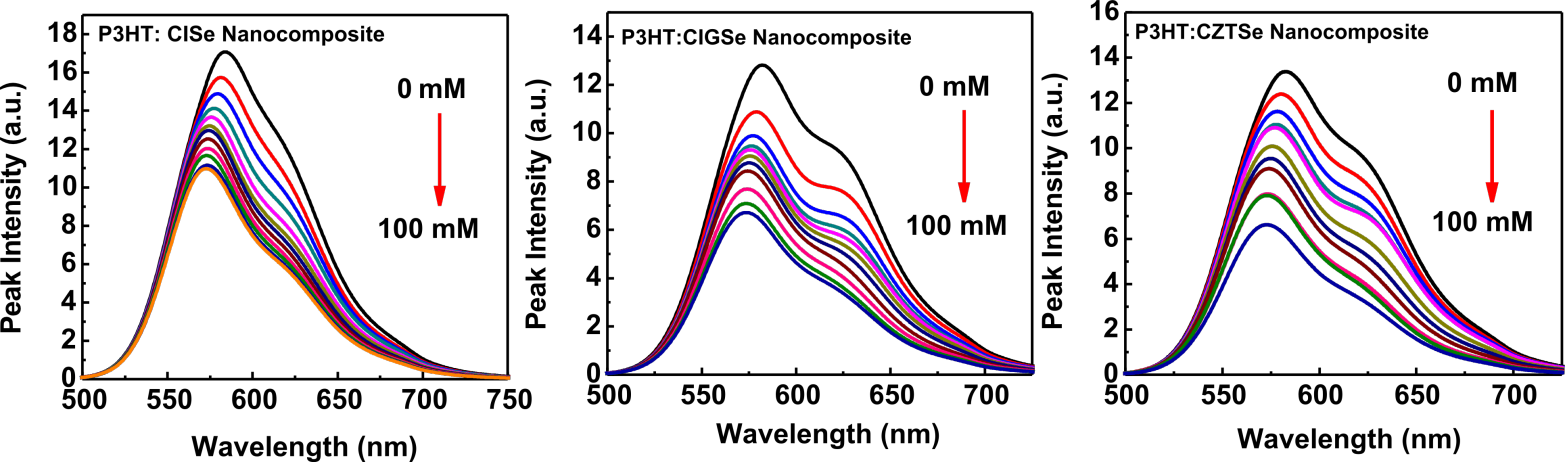
Emission intensity profiles of polymer:chalcopyrite nanocomposites dispersed in toluene (λ_max_ = 580 nm and concentration of chalcopyrites varying from 0–100 mM), (a) P3HT:CISe; (b) P3HT:CIGSe; and (c) P3HT:CZTSe.

Further confirmation of the above observations was provided by investigating the photoluminescence intensities of each of the prepared hybrid nanocomposites of CISe, CIGSe and CZTSe. Emission intensity studies for each of the nanocrystal inks: P3HT were investigated by photoluminescence spectroscopy to study trends of quenching and charge transfer capabilities of each of the synthesized nanocrystals CISe, CIGSe and CZTSe when forming polymer nanocomposites with P3HT. Figure 5a–c shows the emission intensity (λ_max_ = 580 nm) profiles of nanocomposites of CISe:P3HT, CIGSe:P3HT and CZTSe:P3HT dispersed in toluene. As evident in Figure 5a, a more pronounced decrease of the emission intensity of the P3HT:CZTSe composite is observed with an increasing CZTSe concentration compared to P3HT:CISe and P3HT:CIGSe polymer nanocomposites. This corroborates the suggestion that a more efficient electron transfer is taking place between the polymer:CZTSe nanocomposites, that are contributing to the separated electron–hole pairs that ensues recombination non-radiatively.

It is noteworthy that the quenching of the luminescence of P3HT only occurs to some extent even at high concentrations of chalcogenide nanocrystals. This could be mainly attributed to the inability of the nanocrystals to uniformly cling to the polymer matrix so that each of the nanocrystals contributes to a reduction in the PL intensity of the polymer. Additionally, a slight phase segregation may occur in the polymer-nanocomposite of P3HT-chalcogenides, which generally takes place whenever the nanocrystals are dispersed in polymer matrices.

**Figure 6:**
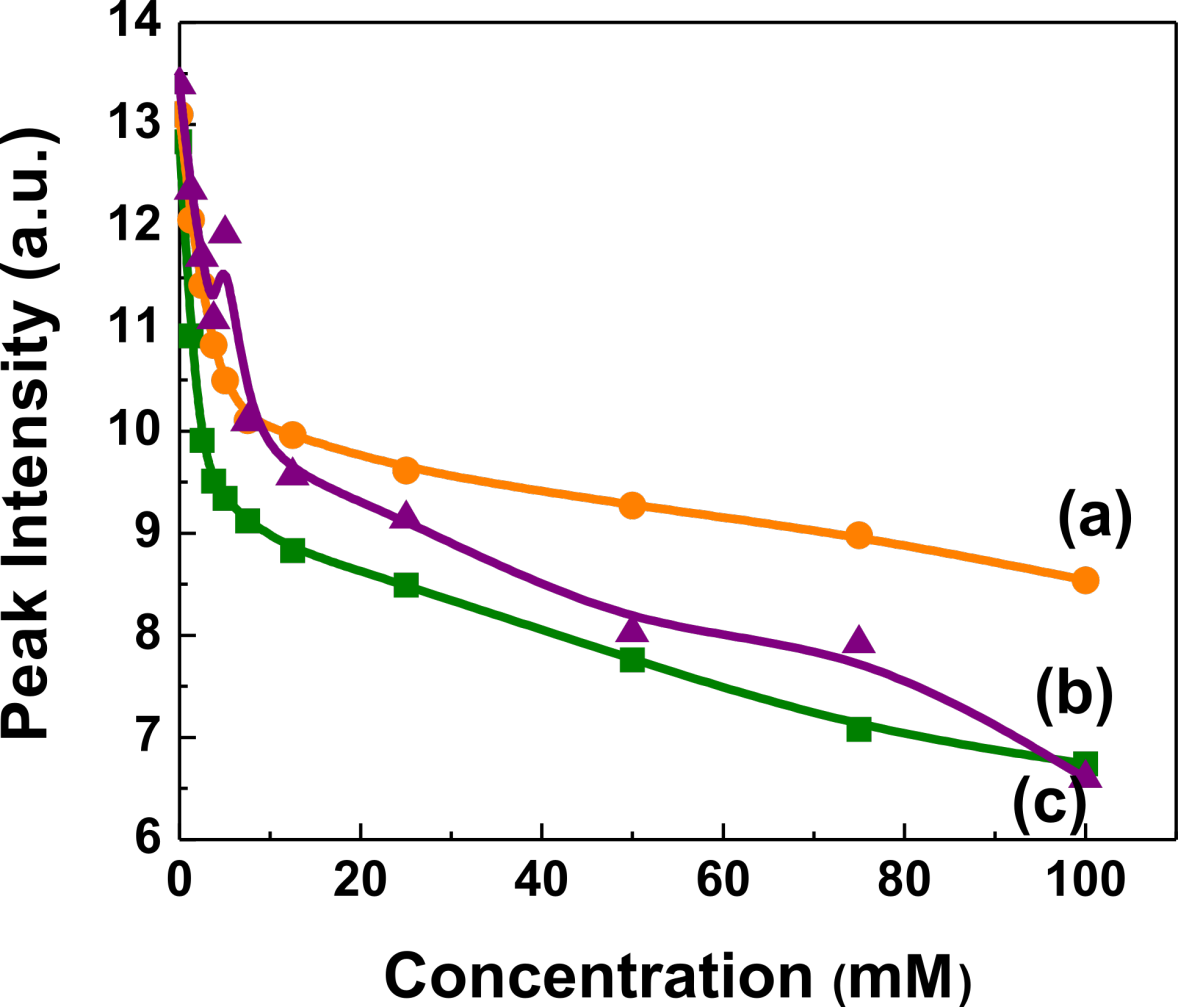
Emission intensity profile of polymer:chalcopyrite nanocomposites as a function of the concentration of chalcopyrite nanocrystals (λ_max_ = 580 nm and concentration of chalcopyrites varying from 0–100 mM). (a) P3HT:CISe; (b) P3HT:CIGSe; and (c) P3HT:CZTSe.

Figure 6 quantitatively demonstrates the reduction in PL intensities for the different polymer nanocomposites CISe, CIGSe and CZTSe, the latter of which exhibited the strongest reduction. The PL quenching rate (ΔPL/PL_initial_; ΔPL ≈ PL_initial_−PL_final_) was 0.35, 0.48 and 0.54 for P3HT:CISe, P3HT:CIGSe and P3HT:CZTSe, respectively.

The mechanism of quenching in polymer based nanocomposites is explained by the Stern–Volmer equation, which is related to the simplest case of collisional quenching of the fluorophore. The Stern–Volmer mechanism [14] is based on the following relation:


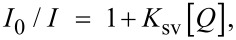


where *I*_0_ and *I* are the fluorescence intensities observed in the absence and presence, of the quencher, respectively, [*Q*] is the quencher concentration and *K*_SV_ is the Stern–Volmer quenching constant.

**Figure 7:**
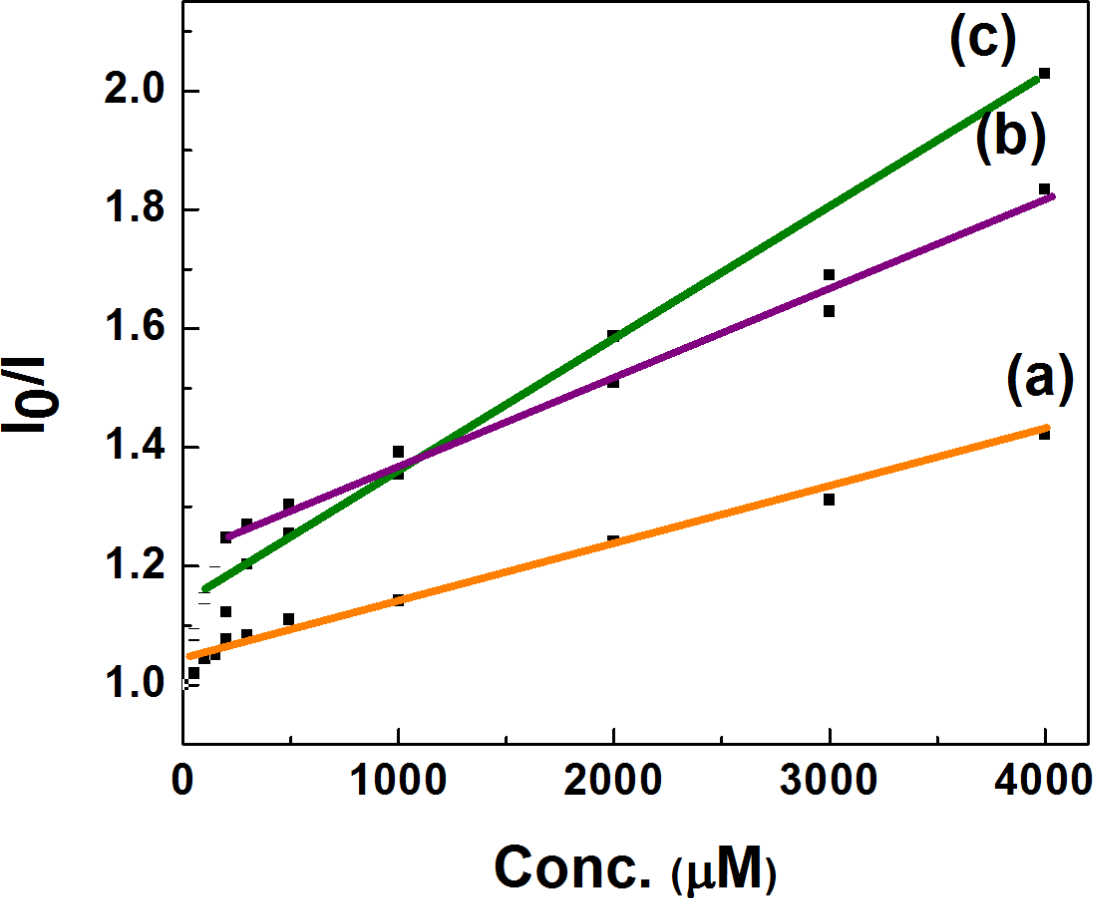
Stern–Volmer plots of (a–c) P3HT:CISe, P3HT:CIGSe, and P3HT:CZTSe nanocomposites.

According to the basic mechanism of the Stern–Volmer quenching phenomenon, the plot of the *I*_0_/*I*-fluorescence intensity ratio should deliver a straight line with gradient of *K*_sv_ [14]. The resulting Stern–Volmer plot is presented in Figure 7 for the fluorescence quenching of CISe, CIGSe and CZTSe nanocrystals prepared by using a colloidal approach and employing TOPO-TOP as the capping agent in each of the individual synthesis of the as-prepared nanocrystals.

However, as depicted in Figure 7a–c deviations from the expected linearity are observed which are more pronounced for small concentrations. This non-linear behavior of the as-prepared chalcogenides nanocrystals could be due to collisional or static quenching. Non-linear Stern–Volmer (S–V) plots are formed when some of the fluorophores are less accessible in comparison to others [15–16]. Thus, the plot of CISe-based nanocomposites points to the existence of two different kinds of fluorophores, one of which is accessible to the quencher to a certain extent, whereas the other is inaccessible, leading to heterogeneous quenching [15–16]. This heterogeneous quenching could be attributed to an insufficient surrounding of the surface of the large-sized nanocrystals by TOPO/TOP ligands, which failed to impose size quantization effects in these nanocrystals resulting in an enhanced size and partial accessibility of the two sets of fluorophores. This facilitated the removal of the loosely bound TOPO/TOP ligand upon washing with the common solvents methanol and toluene and thus aided in the polymer interaction with the surface of the nanocrystals without the utilization of a ligand removal process for the replacement of the bulky TOPO/TOP ligands with the other surface-active groups. From Figure 7a–c, the calculated Stern–Volmer constants *K*_SV_ values for P3HT:CISe, P3HT:CIGSe and P3HT:CZTSe are 0.33 × 10^3^ M^−1^, 1.1 × 10^3^ M^−1^ and 1.3 × 10^3^ M^−1^, respectively. It can be inferred from these values that the P3HT:CZTSe nanocomposites show the highest rate of quenching compared to the hybrids of CISe and CIGSe. *K*_SV_ value for the P3HT:CIGSe polymer nanocomposite precedes the *K*_SV_ value of P3HT:CZTSe nanocomposites, the lowest value was obtained for P3HT: CISe nanocomposites. This indicates an enhanced charge transfer ability of P3HT:CZTSe hybrids compared to the other two according to the PL quenching studies.

**Figure 8:**
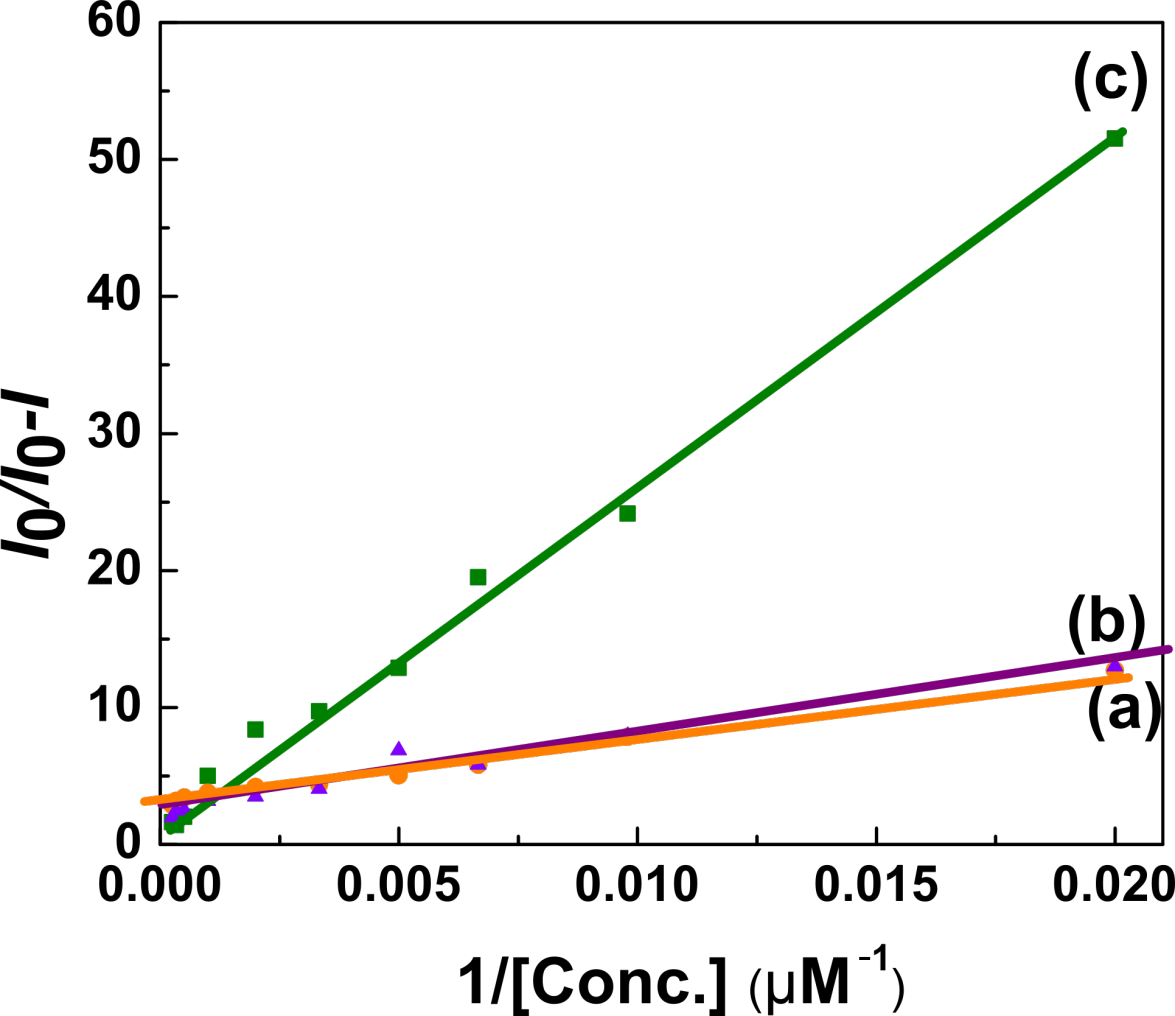
Modified Stern–Volmer plots of (a) P3HT:CISe, (b) P3HT:CIGSe and (c) P3HT:CZTSe nanocomposites.

However, the correspondingly modified (*I*_0_/*I*_0_−*I*) Stern–Volmer plots are shown in Figure 8a–c. Here, we calculated the value of accessible flourophores, *f*_a_, for P3HT:CISe, P3HT:CIGSe and P3HT:CZTSe and the values are found to be ~0.32, ~0.41 and ~0.54, respectively. Among all investigated hybrid nanocomposites of CISe-based chalcogenides, (*f*_a_), the maximum number of accessible flourophores was obtained for P3HT:CZTSe nanocomposites. This implies an utmost efficiency of CZTSe quenchers in intercepting the P3HT fluorophores and rendering them non-fluoroscent compared to the other ones, which results in a better charge transfer capability across P3HT:CZTSe nanocomposites.

**Figure 9:**
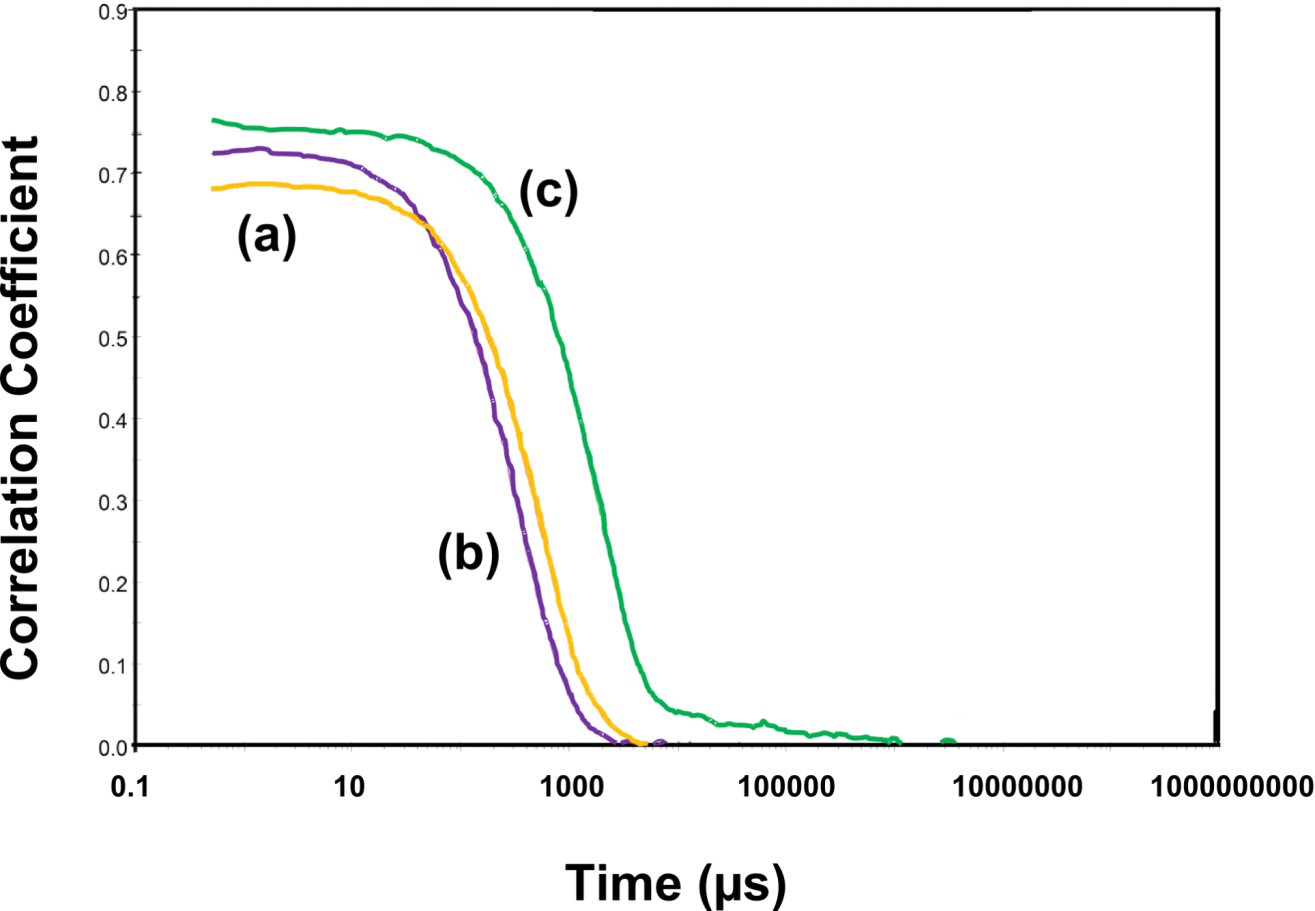
DLS correlation coefficient vs time for (a) P3HT:CISe (b) P3HT:CIGSe and (c) P3HT:CZTSe nanocomposites.

Dynamic light scattering is a method for the determination of particle size distribution and particle agglomerates. It is important to mention that the mean particle diameter (*Z*_average_) for polymer-nanocomposites turns out to be significantly larger than the corresponding values determined by TEM. More specifically, *Z*_average_ is 254 nm (0.123), 470 nm (0.208) and 831 nm (0.271) where values in parenthesis represent polydispersity index (PDI) values. The PDI is an indicator of the broadness of the particle size distribution for P3HT:CISe/CIGSe/CZTSe, respectively. This discrepancy in the size values of nanocomposites determined by DLS and TEM can be understood from the fact that the diameter measured here is actually the hydrodynamic diameter and not the actual diameter of the polymer nanocomposites [17]. Therefore, the size estimation from DLS studies in this work can be ignored. Figure 9 shows a plot of the DLS correlation coefficient vs time for P3HT:CISe/CIGSe nanocomposites. As evident from Figure 9, the correlation decay is steep for P3HT:CISe/CIGSe/CZTSe nanocomposites which implies sample monodispersity [17–18]. However, for the P3HT:CZTSe sample, the decrease of the correlation function is delayed compared to P3HT:CISe/CIGSe nanocomposites. This can be attributed to the incorporation of CZTSe nanocrystals in the polymer matrix which results in the formation of larger crystalline domains due to an increased local order leading to an increased polydispersity and thus a higher PDI value. Inorganic nanocrystals, particularly CZTSe, impart regimentation and stability within the polymer matrix. Thus, the DLS results are in good agreement with other characterization techniques (TEM, PL and light-soaking).

**Figure 10:**
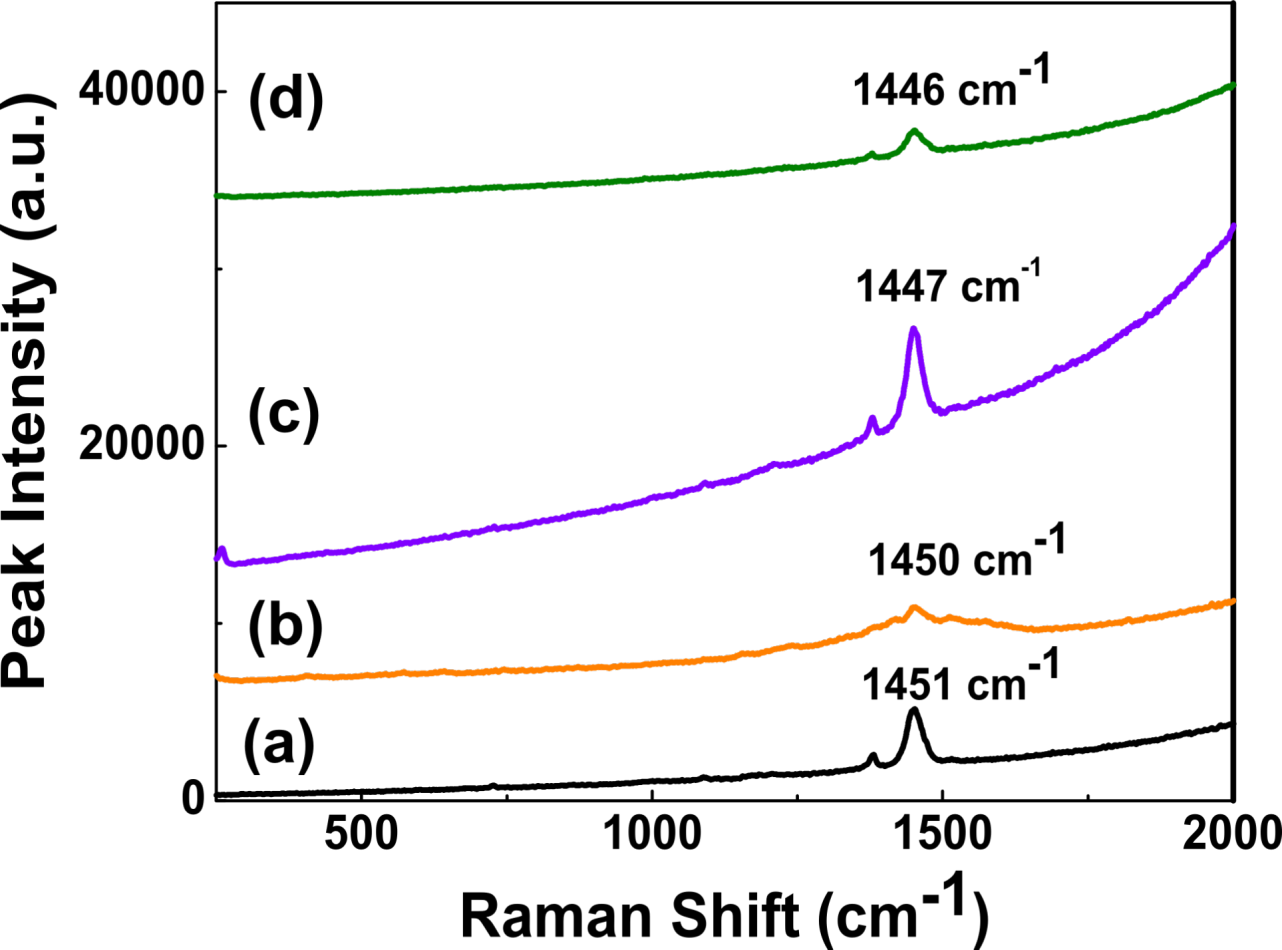
Raman Spectra of (a) P3HT; (b) P3HT:CISe, (c) P3HT:CIGSe, and (d) P3HT:CZTSe nanocomposites.

Raman spectroscopy can be used to probe the structure of polymer(P3HT):CISe/CIGSe/CZTSe nanocomposites since it is sensitive to both the electronic and the vibrational structure of nanocomposites [19]. Figure 10a–d shows the Raman spectra of P3HT and its corresponding nanocomposites. For pure P3HT the Raman peaks at ~1375 cm^−1^ and 1451 cm^−1^ (Figure 10a) are assigned to the C–C skeletal stretching deformation and the C=C ring stretching deformation, respectively [20–21]. However, for P3HT:CISe/CIGSe/CZTSe nanocomposites there is a downward shift in the 1451 cm^−1^ peak position as evident from Figure 10b, which implies an increment in the crystallinity of the P3HT polymer as well as a concurrent extension of the effective conjugation length along the polymer backbone [22]. This downward shift in Figure 10b–d is distinct in case of P3HT:CIGSe/CZTSe (1447 cm^−1^, 1446 cm^−1^) and marginal (~1450 cm^−1^) for P3HT:CISe nanocomposites. The formation of larger crystalline domains due to an enhanced local order could be an artifact of this increased conjugation length. An indication of the ordering of the material can be gauged from the value of the full-width at half maximum (FWHM) of P3HT and its nanocomposites of the C=C stretching deformation (~1451 cm^−1^). This is found to be less for P3HT:CIGSe/CZTSe and significantly more for P3HT:CISe nanocomposites, which indicates a better ordering and hence crystallinity in the former compared to the latter. However, no appreciable Raman features associated with inorganic components, i.e., CISe/CIGSe/CZTSe could be detected, which is in accordance with other studies [23].

## Model

On the basis of the complementary results of TEM/XRD, Raman and PL we propose a model to explore the influence of the crystallinity of the inorganic components (CISe, CIGSe and CZTSe) on the efficiency of charge pair generation and separation at hybrid organic–inorganic semiconductor heterojunctions. The primary requirement in inorganic/organic hybrid solar cells is to blend a high concentration of inorganic nanoparticles into the polymer matrix to form a percolated network where a phase separation on the macroscopic scale should be avoided. When a photon is absorbed by the donor, i.e., the polymer P3HT, the generation of excitons takes place (process 1). The excitons then diffuse to the polymer–chalcopyrite interface where charge separation occurs (process 2). The overall energetic driving force ∆*E* for the electron transfer from the donor to the acceptor depends on the energy difference between the LUMOs of the donor and the acceptor. The subsequent transport of electrons and holes through the donor polymer and acceptor chalcopyrite to their respective electrodes constitutes the current generation (process 3). The transport of the carriers (i.e., electrons and holes) through the donor polymer and acceptor chalcopyrite has to be sufficiently fast to their respective electrodes in order to avoid non-radiative recombination, which can occur at the interface between the two materials and can therefore alter the device photocurrent [24].

**Figure 11:**
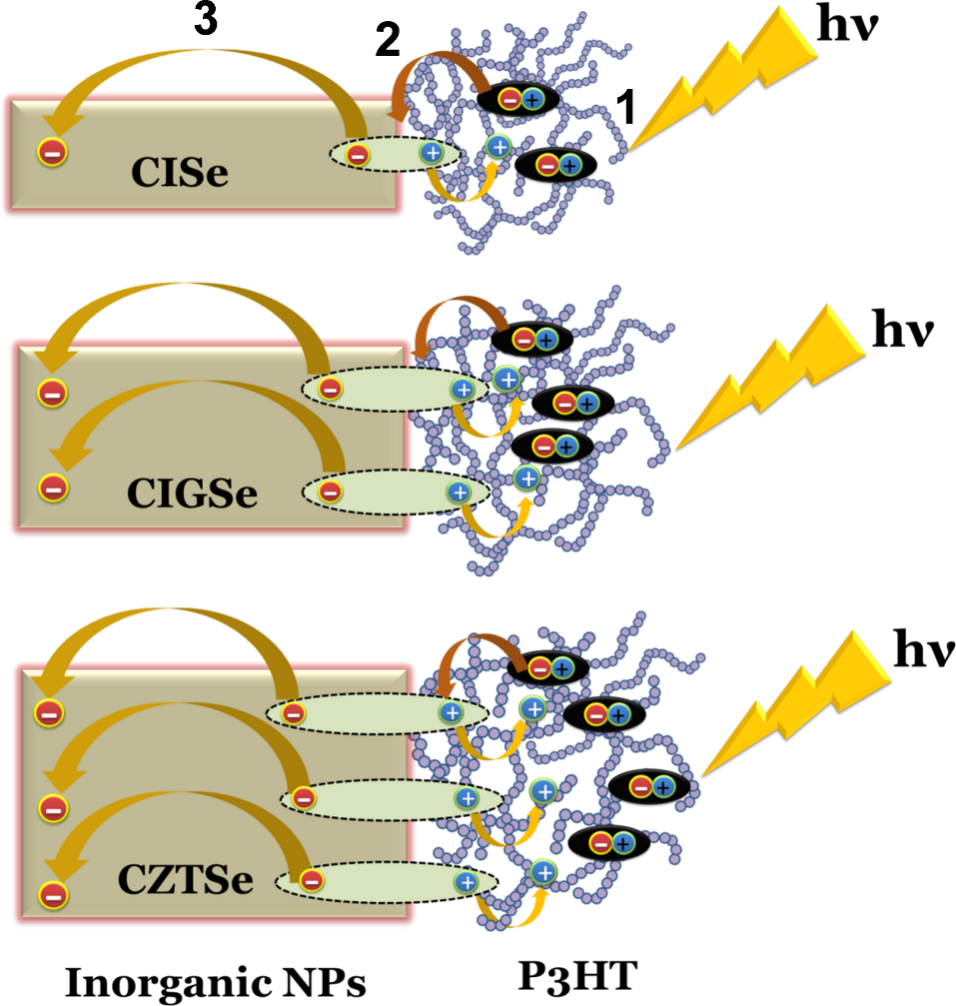
Schematic of the charge generation and separation mechanism. Generation of excitons (1), charge separation (2) and current generation (3) for (a) P3HT:CISe, (b) P3HT:CIGSe and (c) P3HT:CZTSe nanocomposites.

However, after successful exciton dissociation across the D/A interface, electron–hole pairs may remain coulombically bound and therefore susceptible to interfacial recombination. The local junction morphology and the crystallinity of both organic and inorganic components greatly affects such charge-transfer (CT) excitons. In the present case (Figure 11), it is likely that the more crystalline inorganic (acceptor) component (CZTSe) encourages charge delocalization within the CT state resulting in a lower Coulomb binding energy and correspondingly an easier separation of the charges away from the interface. (Figure 11c) compared to the less crystalline components CIGSe and CISe (Figure 11a,b). Furthermore, electrons can migrate longer distances in a crystal of larger crystallite size (CZTSe) due to the formation of a connected network thus leading to a reduction of the volume recombination (Figure 11c).

Here, the higher crystallinity of the inorganic component accounts for an efficient charge separation which can even negate the effect of the interfacial driving energy ∆*E*. The driving energy depends on the difference between the LUMO of the donor (P3HT) and the conduction band of the acceptor (CZTS/CIGS/CIS). For the charge generation and separation across the donor–acceptor interface another possible mechanism of charge transportation could be energy transfer, i.e., the Forster resonance energy transfer (FRET) from the donor to the acceptor after excitation, which results in the generation of an exciton in the acceptor. This mechanism, however, can be ruled out since there is no spectral overlap of the absorption spectra of the acceptor (CISe, CIGSe, CZTSe) and the emission spectra of the donor (P3HT) [25].

## Conclusion

In this work, the mechanism of charge transfer for the ternary (CISe) and quaternary (CIGSe, CZTSe)-chalcopyrites was studied related to the interaction with the regioregular polymer P3HT. Among the discussed set of chalcogenides, CZTSe was found to acquire the most stable stannite/kesterite phase. TEM studies revealed a better tetragonal phase formation with faceted features for CZTSe in comparison to the CISe and CIGSe nanocrystallites, which seem to be characterized by a dominant agglomeration propensity. The profound stability of CZTSe compared to CISe/CIGSe nanocrystallites is also evident from light-soaking studies where an augmentation in crystallinity is observed due to the breaking of weak bonds and a subsequent partial recrystallization. The quaternary chalcopyrites, particularly CZTSe nanocrystals, facilitate an increased crystallinity and thus an increased local order, which provides better steric stability against coagulation, homogeneity and photostability to their respective polymer (P3HT):CZTS composites. The TOPO/TOP–passivated chalcopyrite nanocrystals show evidence of PL quenching in their respective polymer-nanocomposites but with different rates depending upon their degree of crystallization. Due to their higher crystallinity CZTSe nanocrystals show a higher rate of PL quenching, which demonstrates an efficient charge transfer between P3HT:CZTSe compared to the corresponding CISe/CIGSe counterparts. Structural, morphological and optical studies accomplished by various complimentary techniques (TEM, XRD, DLS, PL and Raman) allowed us to compare different hybrid organic (polymer)–inorganic (chalcopyrite) composites. The superior morphology and efficient charge transfer characteristics of the polymer nanocomposite (P3HT:CZTSe) could play a pivotal role for the realization of effective charge separation and transport in hybrid solar cells.

## Experimental

CISe nanocrystals were synthesized by a wet chemical route. More specifically, where a transparent solution formed by dissolving elemental selenium in tri-*n*-octylphosphine (TOP) to prepare a TOPSe complex (TOP) was injected into a solution of an equimolar mixture of InCl_3_ and CuCl_2_ in TOPO at 250–300 °C under an inert gas atmosphere. A color change from straw yellow to deep yellow and finally to black was observed. After cooling to room temperature the reaction mixture was stirred overnight under an argon atmosphere. The black colored solution was then washed with methanol and toluene to remove the excess capping ligand and other organic impurities which may interfere with the properties of the nanocrystals. The nanocrystals were finally dispersed in toluene for further characterizations. Polymer nanocomposites of P3HT:CISe were prepared by adding the known volume of CISe ink to the polymer P3HT and subsequent sonication so that a homogeneous solution is formed. A similar procedure was followed to prepare the CIGSe and CZTSe inks as well as their corresponding polymer nanocomposites with the polymer P3HT. The absorption spectra of the CISe, CIGSe and CZTSe samples were recorded by using a Shimadzu 3101 spectrometer. PL was measured by using a self-assembled system consisting of a two-stage monochromator, a photomultiplier tube with a lock-in amplifier for PL detection, and an Ar^+^ ion laser operating at 488 nm and 5 mW (corresponding to 0.125 W·cm^−2^) for excitation. Dynamic light scattering measurements were carried out by using a Malvern Instrument Nano-S to estimate the nanocrystallite sizes and the size distribution of the chalcopyrite semiconductors.
